# Efficacy and safety of a novel hand dorsum intravenous indwelling needle fixation device in hospitalized pediatric patients: a retrospective controlled study

**DOI:** 10.3389/fped.2025.1616319

**Published:** 2025-11-20

**Authors:** Wanling He, Changchun Lu, Qianyu Qin, Yulin You, Huiyi Tan, Yiming Yao, Shuaijie Guo, Qian Ling, Xinyi Xu

**Affiliations:** 1 Department of Nursing, Guangzhou Red Cross Hospital of Jinan University, Guangzhou, China; 2 Department of Dermatology, Guangzhou Red Cross Hospital of Jinan University, Guangzhou, China; 3 School of Nursing, Guizhou Medical University, Guiyang, China; 4 Department of Pediatric, Guangzhou Red Cross Hospital of Jinan University, Guangzhou, China

**Keywords:** pediatric, peripheral catheterization, nursing care, intravenous catheters, retrospective studies

## Abstract

**Background:**

Proper fixation of dorsal hand intravenous indwelling needles is a key component to ensure the smooth implementation of short-term intravenous therapy for pediatric inpatients. However, traditional fixation methods often face problems such as easy dislodgement and restriction of patient movement, which affect the safety and comfort of treatment. The aim of this study was to evaluate the efficacy and safety of a new dorsal hand intravenous indwelling needle fixation device in clinical applications and to analyze it retrospectively in comparison with traditional fixation methods.

**Methods:**

This was a retrospective controlled study that included a total of 108 pediatric inpatients who were retrospectively divided into Group 1 (*n* = 55) and Group 2 (*n* = 53) based on the type of fixation they actually received. The Group 1 used a new type of fixation device for indwelling needle fixation, while the Group 2 used the traditional fixation method. The total number of indwelling needles used, the average length of time a single indwelling needle was left in place, the comfort score, the incidence of unplanned removal, and the incidence of medical adhesion-related skin injury were compared between the two groups during the hospitalization period.

**Results:**

Compared with the traditional fixation method, the Group 1 with the new fixation device had a significant reduction in the total number of indwelling needles used during hospitalization (*P* = 1.079 × 10^−15^), and a significant prolongation of the average length of time a single indwelling needle was left in place (*P* = 3.136 × 10^−7^). Comfort was significantly improved (*P* = 0.0009), and both the unplanned removal rate and the medical adhesion-related skin injury incidence were significantly reduced (*P* = 6.738 × 10^−5^, *P* = 0.0003, respectively).

**Conclusion:**

The new dorsal hand intravenous needle fixation device can effectively reduce the number of needle changes during hospitalization, prolong the duration of its use, improve patient comfort, and significantly reduce the incidence of unplanned removal and medical adhesion-related skin injury, which has good clinical application value.

## Introduction

In pediatric intravenous therapy, the dorsal hand vein is commonly used for peripheral intravenous catheter (PIVC) due to its superficial location, ease of puncture, and clear visibility (1). However, frequent limb movement and limited cooperation among young children often lead to catheter displacement or unplanned removal (2, 3). These issues directly compromise catheter dwell time, increase the risk of complications, and disrupt the continuity of treatment. Secure and effective catheter fixation is essential to ensure treatment success and minimize adverse events.

Catheter failure remains a significant issue in pediatric care, with reported failure rates ranging from 18.39% to 59.59% depending on age group and clinical setting (2). Reasons for failure include infiltration, dislodgement, occlusion, and phlebitis, all of which are closely related to inadequate fixation (2). Such high failure rates not only increase patient discomfort but also elevate treatment costs and workload for healthcare staff.

Conventional fixation methods, typically involving medical adhesive tape and transparent dressings, have notable limitations in terms of conformability, stability, and comfort, particularly in pediatric patients (4). These traditional methods often fail to adequately accommodate hand movement or the curved surfaces of small pediatric limbs, leading to suboptimal adhesion and higher risk of catheter dislodgement (3, 4). Inadequate fixation may result in unplanned catheter removal, increased need for repeated punctures, and greater procedural burden for both patients and healthcare providers (5).

Furthermore, the immature skin barrier in infants and young children makes them more susceptible to Medical Adhesive-Related Skin Injury (MARSI), which can manifest as maceration, avulsion, or contact dermatitis during dressing application or removal (6). Such injuries may compromise skin integrity and reduce treatment adherence in severe cases (6).

To address these challenges, our team developed a patented fixation device specifically designed for dorsal hand intravenous catheters in infants and young children (Patent No. ZL202021859788.9). The device aims to improve fixation stability and comfort, reduce complication rates, and ultimately enhance the clinical safety and effectiveness of peripheral intravenous therapy. In this study, a retrospective controlled analysis was performed to assess the practical effectiveness and value of this fixation device in clinical applications.

## 2 Methods and materials

### 2.1 Patients selection

Infants and young children who received PIVC therapy during hospitalization in the pediatric department of a tertiary general hospital in Guangzhou were recruited as study participants. The inclusion criteria were as follows: (1) The study was reviewed by the hospital ethics committee and met the appropriate ethical requirements; (2) PIVC performed at the dorsal hand vein; (3) Stable clinical condition without evidence of circulatory disorders. The exclusion criteria included: (1) Presence of skin diseases or infectious conditions; (2) Patients with missing or incomplete relevant medical records.

### 2.2 Novel fixation device

**Figure 1:**
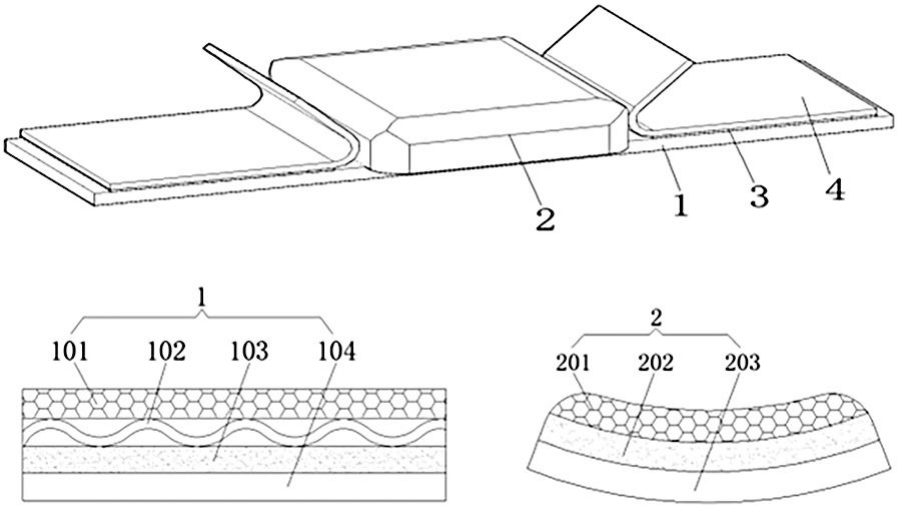
Schematic diagram of the structure of the noval fixation device. 1 fixation strap; 2 palm cushion; 3 transparent adhesive layer; 4 release liner. 101 first breathable layer; 102 absorbent cushioning layer; 103 anti-tear elastic layer; 104 hydrophobic protective layer. 301 second breathable layer; 202 sterile gauze pad; 303 structural base layer made of non-woven fabric.

The novel fixation device is composed of four primary components: a fixation strap, a palm cushion, a transparent adhesive layer, and a release liner. The fixation strap adopts a multi-layer composite structure, consisting of a first breathable layer, an absorbent cushioning layer, an anti-tear elastic layer, and a hydrophobic protective layer. The breathable layer is made of silk fiber with an internal grid-like configuration, providing both softness and ventilation. The absorbent cushioning layer is constructed from sterilized medical cotton, featuring a wavy longitudinal cross-section that increases surface area for enhanced absorption of sweat and fluids. The anti-tear layer is composed of elastic rubber, offering structural support while maintaining flexibility for hand movement. The outer hydrophobic layer is made of polyethylene (PE) film, which is oil-resistant and ensures overall device safety. The palm cushion integrates a second breathable layer, a sterile gauze pad, and a structural base layer made of non-woven fabric. These components work together to improve comfort, promote ventilation, and maintain the structural stability of the fixation system. A transparent adhesive layer ensures secure attachment of the device to the skin, while the release liner facilitates clean and efficient removal without causing skin damage. The detailed structure of the fixation device is illustrated in Figure 1.

### 2.3 Study design

**Figure 2:**
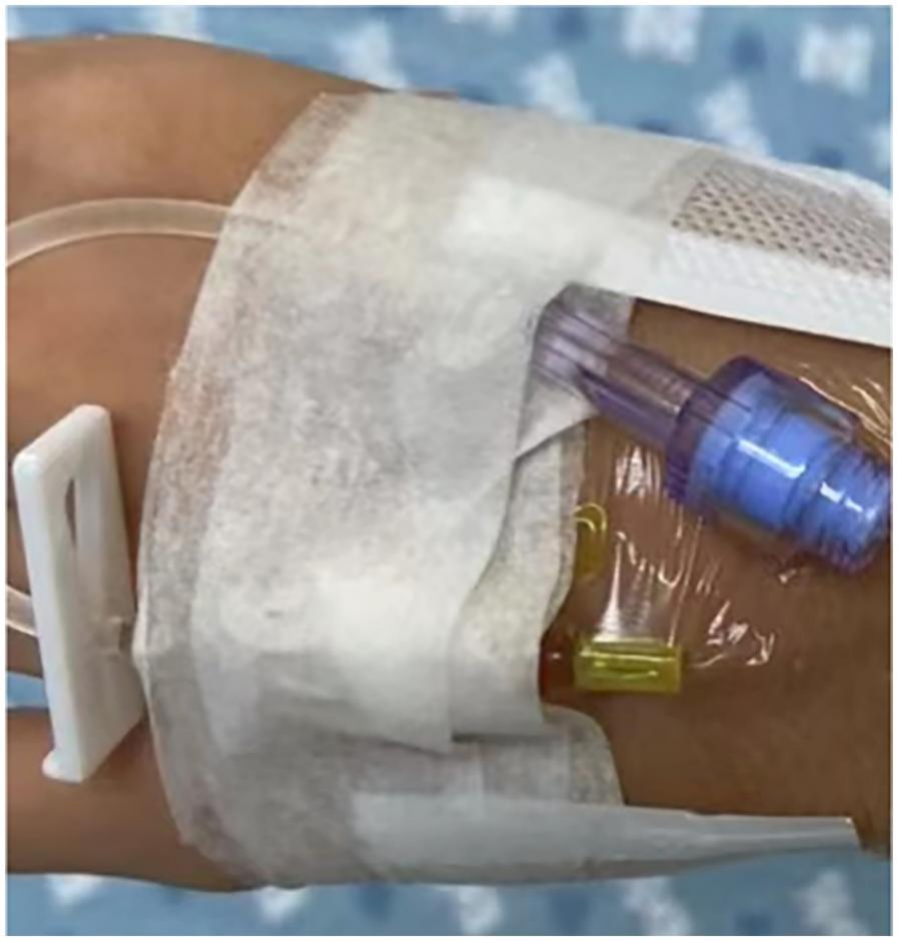
The application of the novel fixation device for hand PIVC securement.

This single-center, non-randomized, retrospective, controlled clinical study aimed to evaluate the efficacy and safety of a novel fixation device for dorsal hand PIVC in infants and young children. The study was conducted between June 2022 and March 2023. A total of 108 eligible infants and young children were included based on predefined inclusion and exclusion criteria. According to the fixation methods documented in the medical records, patients were retrospectively assigned to either the Group 1 (*n* = 55), who received the novel fixation device, or the Group 2 (*n* = 53), who received traditional fixation methods. During hospitalization, all patients underwent PIVC performed by qualified pediatric nurses with over five years of clinical experience. The same type of indwelling needle (24G × 19 mm/Z-G) from a single manufacturer was used across both groups to ensure procedural consistency. Initial fixation was performed using a 3M transparent dressing. In the Group 2, conventional adhesive tape was applied for secondary fixation. In the Group 1, a newly developed fixation device was used according to the following steps: (1) Remove the protective liner from the transparent adhesive layer of the device. (2) Place the palm cushion at the center of the infant's palm, ensuring a snug fit. (3) Attach both ends of the fixation strap to the sides of the palm and secure them using the transparent adhesive. (4) If needed, reinforce the fixation with an additional layer of medical adhesive tape to enhance stability. The application of the novel fixation device for hand PIVC securement is depicted in Figure 2.

All fixation method choices were made by nurses based on a comprehensive assessment of each patient and with full consideration of caregiver preferences, reflecting the best clinical judgment in each case. As the allocation was retrospective and nonrandomized, there is an inherent risk of selection bias, which may affect the generalizability of the results and limit the strength of causal inferences. To mitigate this potential bias, the study took the following measures: (1) baseline characteristics, including age, sex, and underlying conditions, were compared between groups to assess comparability; and (2) any significant differences in potential confounding variables were accounted for in the statistical analyses.

### 2.4 Ethics statement

This study was performed following the principles of the Declaration of Helsinki. The research protocol received approval from the Ethics Committee of Guangzhou Red Cross Hospital of Jinan University (Approval: No.2025-448-07). Written informed consent was obtained from all participants or their legal guardians prior to inclusion. To ensure confidentiality and protect the rights of participants, all data were anonymized.

### 2.5 Data collection

In this study, data on the total number of PIVC used, the average dwell time per catheter, and comfort-related scores were retrospectively extracted from patients' medical and nursing records during hospitalization to evaluate the clinical performance of the novel fixation device. Additionally, the incidence of adverse events, including unplanned removal (UR) of catheter and MARSI, was recorded to assess the safety of the fixation methods. Comfort was assessed using the Chinese version of the Newborn Infant Pain and Discomfort Scale (EDIN), which evaluates five dimensions: facial expression, body movements, quality of sleep, interaction with parents or caregivers, and consolability (7). Each item is scored from 0 to 3, with higher total scores indicating greater discomfort (7). MARSI was evaluated based on the classification criteria developed by the Wound, Ostomy, and Continence Nurses Society (WOCN), which identify whether a skin injury is related to the use of medical adhesive materials (8). The criteria encompass common types of adhesive-related injuries, including mechanical injury, contact dermatitis, maceration, and skin tears (8). Although the EDIN score is a subjective behavioral assessment tool, it objectively captures the comfort level of children who are unable to fully communicate their feelings by evaluating observable behaviors such as facial expressions and body movements. Therefore, it can serve as a valuable supplementary indicator for assessing the effectiveness of indwelling needle fixation.

### 2.6 Statistical analysis

Statistical analyses were performed using SPSS version 26.0 (IBM Corp., Armonk, NY, USA). The normality of continuous variables was assessed using the Shapiro–Wilk test. Normally distributed data were presented as mean ± standard deviation (SD), and between-group comparisons were performed using independent samples *t*-tests. Categorical variables were expressed as counts and percentages (*n*, %), and the chi-square (χ²) test was used for group comparisons. Effect sizes were calculated to evaluate the magnitude of group differences: Cohen's d was used for continuous variables analyzed by *t*-tests, and Cramér's V was used for categorical variables analyzed by chi-square tests. A two-tailed *P*-value of <0.001 was considered statistically significant.

## 3 Results

A total of 108 patients who met the predefined inclusion criteria were retrospectively included in this study, comprising 55 patients in the Group 1 (treated with the novel fixation device) and 53 in the Group 2 (treated with the conventional method). There were no statistically significant differences between the two groups in terms of gender, age, length of hospital stay, or primary diagnosis (*P* > 0.001). Baseline demographic and clinical characteristics of the study participants are presented in Table 1.

**Table 1:** Comparison of demographic characteristics between two groups.

Variables	Group 1 (*n* = 55)	Group 2 (*n* = 53)	χ^2^/*t*	*P*-value
Gender, *n* (%)			0.031^a^	0.861
Female	24 (43.64)	25 (47.17)		
Male	31 (56.36)	28 (52.83)		
Age (month) (mean ± SD)	20.11 ± 11.62	17.17 ± 11.47	1.315^b^	0.191
Length of hospital stay (mean ± SD)	6.91 ± 1.85	7.44 ± 2.05	−1.406^b^	0.163
Diagnostic, *n* (%)			0.981^a^	0.913
Pneumonia	34 (56.36)	36 (67.92)		
Developmental delay disorders	8 (14.55)	8 (15.09)		
Acute upper respiratory tract infection	7 (12.73)	4 (7.54)		
Febrile seizures	4 (7.27)	3 (5.66)		
Urinary tract infection	2 (3.64)	2 (3.77)		

Superscripts indicate the statistical test used. *P*-value <0.001 was considered statistically significant.

a Chi-square test.

b Independent samples *t*-test.

Fixation effectiveness outcome**s** are shown in Table 2. The total number of PIVCs used during hospitalization was significantly lower in Group 1 (1.74 ± 0.44) compared with Group 2 (2.98 ± 0.76) (t = −9.922, *P* < 0.001), with a very large effect size (Cohen's d = 2.01). The average catheter dwell time was significantly longer in Group 1 (75.22 ± 19.36 h) than in Group 2 (56.35 ± 16.26 h) (t = 5.470, *P* < 0.001), with a large effect size (Cohen's d = 1.05). Comfort scores assessed by the EDIN scale were significantly better (lower) in Group 1 (1.16 ± 1.57) than in Group 2 (2.34 ± 1.97) (t = −3.420, *P* < 0.001), indicating a medium-to-large effect size (Cohen's d = 0.66).

**Table 2:** Comparison of immobilization effectiveness between the two groups.

Variables	Group 1 (*n* = 55)	Group 2 (*n* = 53)	*t*-value	*P*-value	Cohen's d
PIVC’ total number	1.74 ± 0.44	2.98 ± 0.76	−9.922	<0.001	2.01
PIVC’ average of dwell time (h) (mean ± SD)	75.22 ± 19.36	56.35 ± 16.26	5.470	<0.001	1.05
EDIN (mean ± SD)	1.16 ± 1.57	2.34 ± 1.97	−3.420	<0.001	0.66

The above data were analyzed using independent two-sample *t*-tests. Effect sizes (Cohen's d) were calculated to quantify the magnitude of differences between groups, with larger values indicating stronger effects. A *P*-value <0.001 was considered statistically significant.

Fixation safety outcomes are presented in Table 3. Accidental removal occurred in 2 patients (3.64%) in Group 1 vs. 19 patients (35.85%) in Group 2 (χ² = 15.883, *P* < 0.001), with a moderate association (Cramér's V = 0.39). Moreover, no cases of MARSI were observed in Group 1, compared with 13 cases (24.53%) in Group 2 (χ² = 13.108, *P* < 0.001), also with a moderate association (Cramér's V = 0.36).

**Table 3:** Comparison of immobilization safety between the Two groups.

Variables	Group 1 (*n* = 55)	Group 2 (*n* = 53)	χ^2^	*P*-value	Cramér's V
Accidental removal, *n* (%)	2 (3.64)	19 (35.85)	15.883	<0.001	0.39
MARSI, *n* (%)	0 (0)	13 (24.53)	13.108	<0.001	0.36

The above data were analyzed using Chi-square tests. Effect sizes (Cramér's V) were calculated to quantify the strength of association between groups, with larger values indicating stronger effects. A *P*-value <0.001 was considered statistically significant.

## 4 Discussion

This study evaluated the clinical effectiveness and safety of a novel PIVC fixation device for pediatric use. Compared with conventional fixation methods, the device significantly improved catheter stability, prolonged indwelling time, enhanced patient comfort, and reduced the incidence of UR and MARSI, demonstrating considerable potential for integration into routine pediatric care. Meanwhile the device has been granted a utility model patent by the China National Intellectual Property Administration (Patent No. ZL202021859788.9), underscoring its originality and technical distinctiveness. A literature search revealed no comparable clinical studies or widely available commercial products with the same structural design.

Despite these promising findings, the retrospective and nonrandomized design of this study necessitates cautious interpretation. In routine clinical practice during the study period, the choice of fixation method was influenced by pragmatic considerations: nurses tended to select the novel device for children who were more active, anticipated to require longer catheter retention, had experienced previous failure with conventional fixation, or had skin conditions better suited to the new device. Conversely, the conventional method was more often chosen for children with shorter expected retention times, milder illness, or when caregivers preferred it. This allocation pattern resulted in the novel device group containing more patients with poor vascular access or greater mobility—factors that inherently increase the risk of complications. It was worth noting that baseline demographic and clinical characteristics (age, sex, primary diagnosis, length of hospital stay) did not differ significantly between groups. In other words, the novel device was tested under more stringent clinical conditions, yet still exhibited significant advantages, suggesting that the observed benefits may represent a conservative estimate of its true effect.

In China, approximately 70% of hospitalized children require intravenous fluid therapy (2, 9). The PIVC is a standard short-term infusion device that plays a crucial role in ensuring treatment continuity and patient comfort. However, achieving effective fixation that both stabilizes the catheter and protects the delicate skin of infants and young children remains a key challenge in pediatric care.

Evidence from previous investigations indicates that the application of innovative securement approaches—such as tissue adhesives (TA) and integrated securement dressings (ISD)—has been associated with lower PIVC failure rates in both pediatric and adult cohorts (10–12). In emergency department settings, a randomized clinical trial reported that TA decreased PIVC failure within 48 h from 27% to 17% (*P* = 0.02) (11). Conversely, a large-scale randomized controlled study among hospitalized adults (*n* = 1,807) did not replicate this benefit (12), suggesting that the clinical environment may influence the efficacy of catheter securement. In this study, the PIVC average of dwell time in the novel fixation device group (Group 1) was 75.22 ± 19.36 h, approximately 19 h longer than in the conventional fixation group (Group 2) (56.35 ± 16.26 h). This result is consistent with previous findings showing that PIVC dwell time can be maintained for 3–4 days when scientifically designed fixation devices are used (13, 14). The extended retention time observed with the new fixation device may result from its integrated structural support and biocompatible adhesive layer, which collectively reduce micro-movements and mechanical shear stress at the insertion site. These design optimizations help maintain catheter patency and minimize the likelihood of dislodgement. Moreover, appropriate catheter maintenance and close monitoring during hospitalization further contribute to prolonged retention.

Patient comfort is another critical dimension in pediatric intravenous therapy (15). During the fixation of intravenous indwelling needles in pediatric patients, patient comfort plays a pivotal role in influencing their cooperation and emotional regulation (15, 16). An optimized fixation method that improves comfort can mitigate adverse behaviors such as struggling or pulling, which are often elicited by discomfort, thereby reducing the incidence of catheter displacement and unplanned removal (17). In this study, patients in Group 1 reported significantly higher comfort scores, likely owing to the device's soft, breathable materials and ergonomic contour that conform to pediatric skin physiology. Such characteristics not only alleviate local irritation and discomfort but also allow for greater mobility, thereby improving both patient tolerance and caregiver satisfaction.

Regarding fixation safety, previous studies have reported that the failure rate of PIVC in pediatric patients can reach up to 36%, with UR being the primary cause (12, 18). In this study, the UR rate in the Group 2 was 35.85%, consistent with previous findings and highlighting the limitations of traditional tape-and-dressing fixation methods in children. In contrast, the UR rate in the Group 1 was only 3.64%, representing a statistically significant reduction. This finding aligns with prior evidence that scientifically designed fixation devices can reduce catheter displacement risk by up to 50% (19). The markedly lower UR rate in the new device group may be explained by its dual advantages: enhanced comfort, which increases patient tolerance and reduces active removals due to discomfort, and improved fixation effectiveness, which stabilizes the catheter and minimizes passive dislodgement.

MARSI remains a common complication in pediatric intravenous therapy. Previous studies reported that up to 80.4% of these injuries are caused by traditional adhesive materials (19, 20). The removal of adhesive materials has been reported to detach up to 70%–90% of an infant's epidermal layer, and repeated applications can lead to even deeper skin damage (21–23). This occurs because the bonding strength between adhesive tape and the skin surface often exceeds the cohesion between the epidermal layers themselves. In line with our study, 28.30% of the patients in Group 2 experienced MARSI while no MARSI events occurred in the Group 1. This protective effect is likely attributable to the device's design that minimizes direct contact between skin and adhesive, combined with a low-allergy adhesive layer and pressure-distributing structure, effectively reducing the risk of epidermal injury.

## 5 Limitations

This study has several limitations that should be considered when interpreting the findings. First, the retrospective, non-randomized design may introduce selection bias. Second, the sample was drawn from a single center, which may limit the generalizability of the results. To address these limitations and further validate the clinical utility of this novel fixation device, future studies should consider prospective, randomized controlled trials across multiple centers. In addition, cost-effectiveness analyses and long-term follow-up investigations would be valuable to assess its broader clinical applicability and economic impact.

## 6 Conclusion

This study offers the first clinical evidence supporting the application of a patented hand dorsum intravenous catheter fixation device specifically designed for pediatric patients. The device demonstrated notable advantages in reducing unplanned removal and skin-related complications while enhancing catheter dwell time and patient comfort. Its user-friendly design and potential to improve clinical outcomes highlight its value in pediatric intravenous therapy. Future large-scale, multicenter randomized trials are needed to further substantiate these findings and support wider clinical adoption.
